# Comparison of Vault Measurements Using a Swept-Source OCT-Based Optical Biometer and Anterior Segment OCT

**DOI:** 10.3389/fmed.2022.865719

**Published:** 2022-06-23

**Authors:** Yali Du, Chuang Jin, Shengjie Yin, Geng Wang, Qian Ma, Yuancun Li, Binyao Chen, Hongxi Wang, Kunliang Qiu, Mingzhi Zhang

**Affiliations:** Joint Shantou International Eye Center of Shantou University, The Chinese University of Hong Kong, Shantou, China

**Keywords:** IOLMaster700, ICL, vault, AS-OCT, C-ICL, ACD

## Abstract

**Background:**

To newly describe the vault measurement by using a widely used swept-source OCT-based optical biometer (IOLMaster700) and accessd the accuracy of vault measurement.

**Methods:**

This was a retrospective, cross-sectional study. All patients underwent implantable Collamer lens (ICL) implantation surgery without complications. IOLMaster700 and AS-OCT analyses were conducted for each eye on the same day in the same condition. Measurements of anterior chamber depth (ACD), corneal-ICL (C-ICL), and vault values were made and recorded. The repeatability of the IOL Master700 measurements was quantified based upon intraclass correlation coefficient (ICC) values. Correlations between IOL Master700 and AS-OCT measurements made with these different analytical approaches were assessed. The agreement of instruments was evaluated using Bland-Altman plots.

**Results:**

The IOLMaster700 instrument yielded highly reliable measurements of vault, C-ICL, and ACD (*ICC* = 0.996, 0.995, 0.995, respectively). Vault, C-ICL and ACD values as measured using the IOLMaster700, was slightly smaller than that measured via AS-OCT, but these differences were not significant (*p* = 0.652*, p* = 0.121 and *p* = 0.091, respectively). The vault, C-ICL, and ACD measurements by these two instruments were strongly correlated (*r* = 0.971, *r* = 0.944, and *r* = 0.963, respectively; all *p* < 0.001). The 95% limits of agreement for vault, C-ICL, and ACD measurements between the two devices were−0.08 to 0.08 mm,−0.14 to 0.11 mm, and−0.13 to 0.10 mm, respectively.

**Conclusions:**

The IOLMasrer700 can measure implanted ICL vault with a high degree of accuracy and repeatability. Good correlations and agreement were observed between IOLMaster700 and AS-OCT in measuring vault, C-ICL, and ACD measurements.

## Introduction

Implantable Collamer lens (ICL) implantation is increasingly common as a safe and efficacious corrective treatment for myopia (1–4). Achieving success when performing ICL implantation necessitates the accurate prediction of refraction and postoperative ICL lens vault, which correspond to the distance between the crystalline lens anterior surface and the ICL posterior surface. Vault is a critical parameter associated with safety outcomes related to ICL implantation, as insufficient vault can result in negative events including pupillary block, pigment dispersion, cataract development, lens exchange, corneal endothelium cell loss, or increased intraocular pressure (IOP) (5–7).

Broadly speaking, an acceptable vault value is between 250μm and 1,000μm (8) (or 750μm) (9) in some reports. ICL vault measurements are commonly performed via anterior segment optical coherence tomography (AS-OCT), ultrasound biomicroscopy (UBM), or with a Pentacam instrument (Oculus,Germany), but no gold standard measurement strategy exists. Indeed, ICL vault values can differ significantly among these different measurement techniques (10).

The IOLMaster700 instrument (Carl Zeiss Meditec AG, Germany) is a recently developed swept-source OCT-based optical biometer that can measure parameters including axial length (AL), anterior chamber depth (ACD), central corneal thickness (CCT), and lens thickness (LT) with a tunable 1,055 nm laser, in addition to permitting visualization and OCT imaging of the full eye (11, 12). Despite the widespread use of the IOLMaster700, it cannot measure vault after ICL implantation directly. In this article, we employed the ImageJ software to measure ICL vault using an IOLMaster700 instrument and evaluated the accuracy of these measurements.

No prior studies have reported on vault measurements made using the IOLMaster700. Therefore, we used this instrument to scan ICL implantation anterior segment parameters in order to evaluate the accuracy of vault measurement by using this new technology.

## Methods

### Subjects and Methods

All patients undergoning ICL (Visian V4c, STAAR Surgical, Switzerland) implantation at Joint Shantou International Eye Center of Shantou University and The Chinese University of Hong Kong (JSIEC) were consecutively recruited from September 2020 to December 2021. The current study was approved by the local clinical research ethics committee (No. EC20200609) and was conducted in accordance with the Declaration of Helsinki. The requirement for informed consent was waived due to the retrospective design of the current study. All patients were evaluated during regularly scheduled follow-up.

### ICL Calculation and Size Selection

Horizontal white to white (WTW) and ACD values were measured by corneal topography (Orbscan II; Bausch &Lomb, Rochester, NY, USA). AS-OCT (CASIA 1/CASIA 2, Tomey Corp.Japan) was conducted to measure angle to angle (ATA) and crystalline lens rise (CLR). Cliary sulcus was performed by UBM. Ocular biometric measurements were completed by using the IOLMaster700 instrument. ICL power calculations were completed by STAAR Co using the modified vertex formula, and ICL sizes were chosen as per the NK formula (13).

### Surgical Approach

Two experienced surgeons (ZMZ,WG) performed all surgical procedures for each subject. A slit-lamp was used to preoperatively mark the zero horizontal axis for toric ICL implantation. Following the application of topical anesthesia, a viscoelastic substance was applied to the anterior chamber. An injector cartridge (STAAR Surgical) was then used to insert aV4c ICL via a 3.0 mm clear corneal incision such that the ICL was positioned within the posterior chamber. Following correction of the ICL position, the viscoelastic substance was replaced with a balanced salt solution. No patients experienced any surgery-related complications. Postoperatively, patients were administered 0.5 levofloxacin topically and steroidal medications four times per day over a three-week period, with doses being gradually reduced.

### Postoperative Measurement

Postoperative anterior segment imaging was performed with both the AS-OCT and IOLMaster700 instruments on the same day under identical lighting conditions in the same room. Anterior chamber depth (ACD), corneal-ICL (C-ICL), and vault were documented for further analysis. Vault was defined as the distance from the crystalline lens anterior surface to the ICL posterior surface. ACD, the distance from the crystalline lens anterior surface to the corneal endothelium, was automatically measured using the built-in software. C-ICL was the distance from the ICL anterior surface to the corneal endothelium.

**Figure 1:**
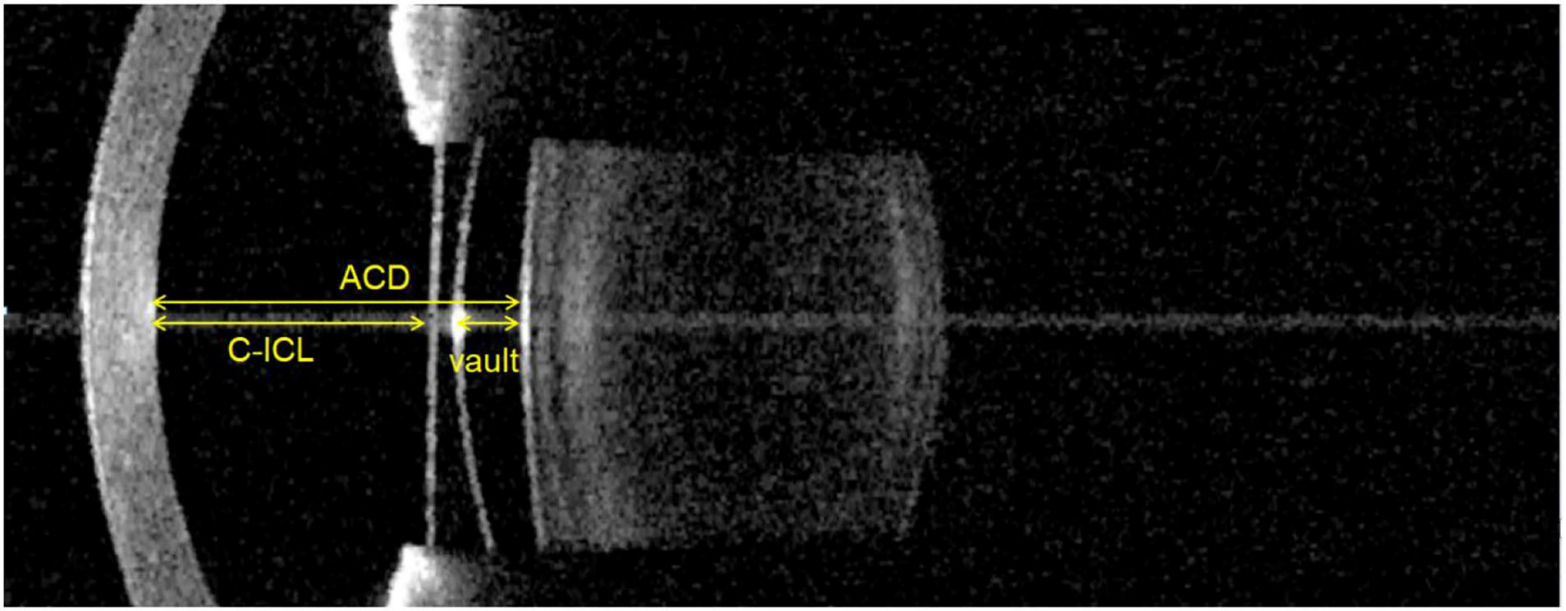
Vault, C-ICL, and ACD measurements made using the IOL Master 700. Vault: Distance between the anterior surface of the crystalline lens and the posterior surface of the ICL. C-ICL: Distance between the corneal endothelium and anterior surface of the ICL. ACD: Distance between the corneal endothelium and the anterior surface of the crystalline lens.

IOLMaster700 measurements were made using a phakic intraocular lens (P-IOL) pattern and B scans at 0 degrees. The parameters of the IOL Master 700 were measured by the same doctor using the ImageJ software (http://rsb.info.nih.gov/ij; National Institutes of Health, Bethesda, Maryland, USA). First, centering on the pupil axis, the corneal thickness was taken as the benchmark. On the pupil axis, anterior chamber depth (ACD), C-ICL and vault were measured by IOL Master 700 images (Figure 1) and converted pixels to millimeters. To assess the repeatability of measurements, a single doctor measured these parameters three times.

AS-OCT (CASIA 1/CASIA 2, Tomey Corp., Japan) measurements were also conducted, with horizontal median images being captured for subsequent analyses. Measurements were made with an on-screen calibration system.

### Statistical Analysis

Based on a previous study that employed an AS-OCT-based measurement starategy, the average vault was 0.64 mm, with a standard deviation (SD) of 0.25 mm (10). At a significance (α) level of 0.05 and a power (β) of 80%, a sample size calculation indicated that a minimum of 16 subjects would be required to give 80% power at a 5% two-sided significance level to detect a 5% mean difference. The repeatability of the IOL Master700 measurements was quantified based upon intraclass correlation coefficient (ICC) values. The differences between instruments were analyzed by paired *t*-test, while correlations between the two instruments were assessed using Pearson correlation coefficient (*r*) values. Bland and Altman plots were employed to assess the agreement of vault, C-ICL, and ACD measurements between different devices. Measurement variability was evaluated using 95% limit of agreement values (95% LOA = mean agreement). *P* < 0.05 was the significanct threshold.

## Results

In total, this study enrolled 82 eyes of 43 patients (mean age: 26.03 ± 4.05 years, range: 19 to 36); 19 (44.19%) male, 24 (55.81%) female). The mean follow-up time was 5.04 ± 5.20 months (range: 1–12 months). The baseline characteristics of the study population were shown in Table 1. A non-toric ICL was implanted in 29 eyes (35.4%), while 53 eyes (64.6%) underwent toric ICL implantation.

**Table 1:** Baseline patient characteristics.

**Parameters**	
Age	26.03 ± 4.05 (19 to 36)
Sex (female, male)	24/19
Manifest refractive sphere(D)	−10.41 ± 3.17 (−4.75 to −19.5)
Manifest refractive cylinder(D)	−1.98 ± 1.34 (0 to −5.5)
Axial length (mm)	28.01 ± 1.71 (24.89 to 31.98)
TICL / ICL (*n*)	53/29
Follow-up (months)	5.04 ± 5.20 (range 1–13 months)

*D, diopter; n, number; mm, millimeter*.

### Assessment of the Repeatability of Vault Measurements by Using the IOLMaster700

ICC results in Table 2 demonstrated the repeatability of vault measurements, C-ICL measurements, and ACD measurements obtained using the IOLMaster700 instrument, consistent with the high reliability of this technology as a tool for measuring these three parameters (*p* < 0.001).

**Table 2:** Repeatability of IOL Master 700 measurements of vault, C-ICL, and ACD.

	**ICC**	**95%lower**	**95%upper**	***p-*value**
Vault	0.996	0.993	0.998	<0.001
C-ICL	0.995	0.991	0.998	<0.001
ACD	0.997	0.995	0.999	<0.001

### Comparison of IOL Master700 and AS-OCT Measurements

Table 3 demonstrates the comparison of parameters measured using IOLMaster700 and AS-OCT approaches. Vault, C-ICL and ACD values as measured using the IOLMaster700 were slightly smaller than measurement with AS-OCT, but there were no significant differences (*p* = 0.652*, p* = 0.121 and *p* = 0.091, respectively).

**Table 3:** Comparison of parameters measured using the IOL Master700 and AS-OCT.

	**IOLMaster700**	**AS-OCT**	***T***	***p-*value**
Vault (mm)	0.58 ± 0.17	0.59 ± 0.18	−0.453	0.652
C-ICL (mm)	2.38 ± 0.18	2.40 ± 0.19	−2.574	0.121
ACD (mm)	3.22 ± 0.22	3.24 ± 0.22	−2.675	0.091

### Correlations and Agreement Between IOL Master700 and AS-OCT Measurements

**Figure 2:**
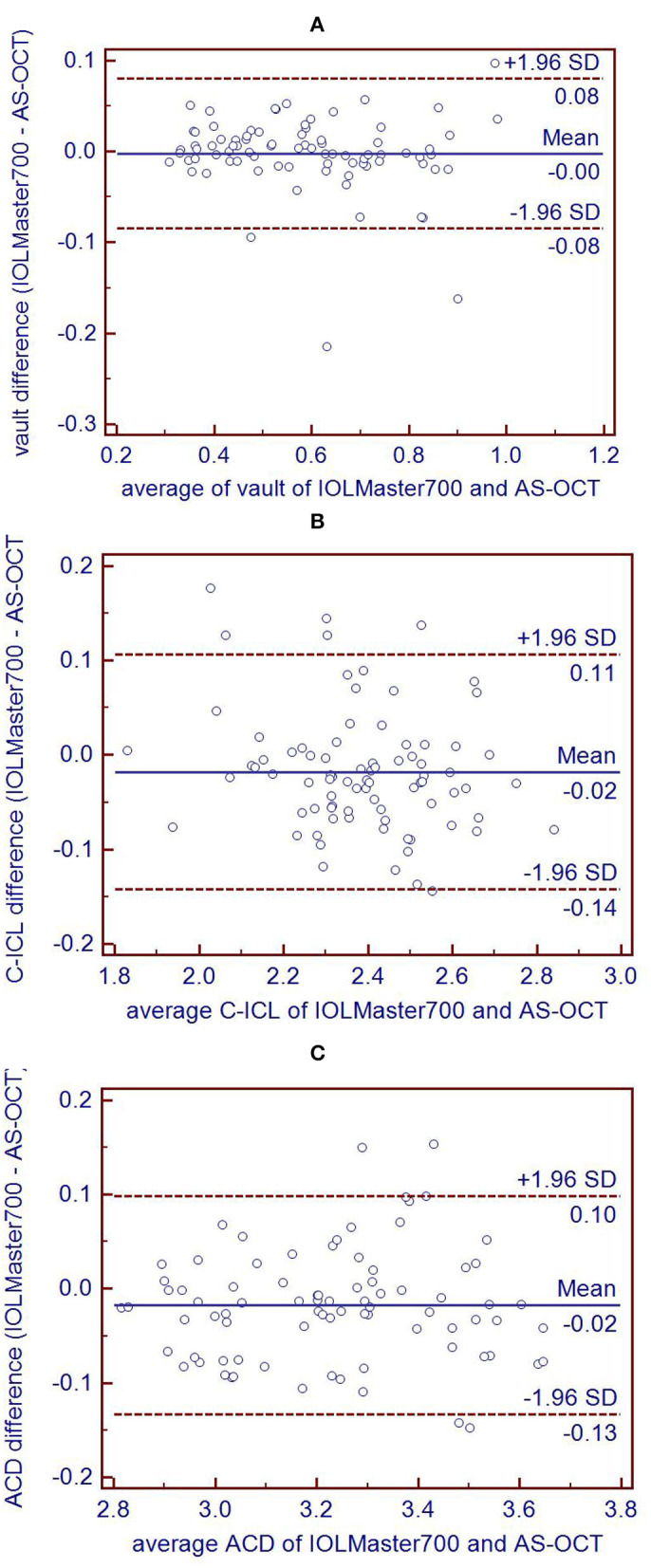
Bland-Altman plots comparing the level of agreement between IOL Master 700 and AS-OCT measurements of vault **(A)**, C-ICL **(B)**, and ACD **(C)**. The vertical axis represents the difference between these measurements and the horizontal axis shows the corresponding mean value. The 95% LoA is indicated using dashed lines, and the middle bold line represents the mean difference between these measurements.

As shown in Table 4, there were significant correlations between the vault, C-ICL, and ACD values measured via the IOLMaster700 and AS-OCT (*r* = 0.971, *r* = 0.944, and *r* = 0.963, respectively; *p* < 0.001). Good agreement was observed between the measurement values obtained from the IOLMaster700 and AS-OCT. Bland-Altman analysis plots indicated that the 95% limits of agreement for vault, C-ICL, and ACD measurements were−0.08 to 0.08 mm,−0.14 to 0.11 mm, and−0.13 to 0.10 mm between these two devices, respectively (Figure 2).

**Table 4:** Agreement and correlations among parameters measured using the IOL Master700 and AS-OCT.

	**IOL Master700**	**AS-OCT**	**Mean of difference (95%LoA)**	**Coefficient (*r*)**	***P-*value**
Vault	0.58 ± 0.17	0.59 ± 0.18	0.00 (−0.08 to 0.08)	0.971	<0.001
C-ICL	2.38 ± 0.18	2.40 ± 0.19	−0.02 (−0.14 to 0.11)	0.944	<0.001
ACD	3.22 ± 0.22	3.24 ± 0.22	−0.02 (−0.13 to 0.10)	0.963	<0.001

*Pearson (r), Pearson correlation coefficient; LoA, limit of agreement*.

## Discussion

One of the most important parameters to be assessed after ICL implanted was the measurement of vault (14). Studies had been reported on complications after ICL implantation associated with vault (5–7). Low vault was a risk factor of formation of cataract. High vaults can produce an narrow of the anterior chamber angle at risk of developing increased intraocular presssure.AS-OCT (CASIA) was widely used for vault measurement after ICL implantation. The CASIA system is a swept source OCT that uses a wavelength of 1,310 nm, providing an axial resolution of 10μm or less and a scan speed of 55,000 A-scans per second (14). It was an effective instrument for measuring Vault.

Herein, we used the IOLMaster700 instrument to quantify ICL implantation anterior parameters. IOL Master 700 is the most frequently used device in optical biometry measurements and provides precise results of biometry parameters of the anterior eye segment, currently considered the so-called “gold standard” for axial length measurement (15). This non-contact high-resolution biometric instrument utilizes a tunable laster centered at 1,055 nm to generate B scans that enable operators to quantify ocular data (16). The IOLMaster700 can perform 44 mm scans at 22μm resolution (12), and can detect crystalline lens features including decentration or tilt as the generated B-scans are shown as full-length OCT images of anatomical details along longitudinal sections through the full eye (17). At present, this instrument cannot directly measure postoperative lens vault values, and there have been no prior publications regarding the use of the IOLMaster700 for measurements made in the context of ICL implantation. We therefore examined the repeatability of vault, C-ICL, and ACD measurements made using the IOLMaster700 and found it to exhibit good repeatability. We then compared correlations between the IOLMaster700 and AS-OCT approaches to measuring vault, C-ICL, and ACD.

There are several approaches available that can be employed to measure vault height after ICL surgery including AS-OCT, Pentacam, and UBM. However, no gold standard approach has emerged to date (10). UBM can facilitate precise analyses of anterior segment structures. However, it is an invasive examination, it cannot be used during early periods after ICL surgery. Furthermore, vault measurements made via UBM are prone to measurement errors. AS-OCT and Pentacam are more commonly used for measuring vault, and prior reports have shown that these measurement values varyed from instrument to instrument (10, 18).

In our study, vault, C-ICL and ACD values as measured using the IOLMaster700, was slightly smaller than measurement with AS-OCT, but these differences were not significant. This may be due to differences in device resolution or scan location. Differences in the refractive indices of ICL materials and the humor can also impact these measurements. Even so, we observed very strong correlations between AS-OCT and IOLMaster700, consistent with clinically acceptable differences between these two devices.

There were at least two limitations to the present study. Firstly, we used the image analysis software ImageJ to calculate these values, potentially introducing measurement error. Secondly, we did not compare vault, C-ICL, and ACD measurements with other devices, such as Pentacam or UBM.

In conclusion, the repeatability of vault, C-ICL, and ACD measurements made using the IOLMaster700 was high. Good correlation and agreement were found between IOLMaster700 and AS-OCT measurements.

## Data Availability Statement

The original contributions presented in the study are included in the article/supplementary material, further inquiries can be directed to the corresponding author/s.

## Author Contributions

Conception and design: MZ and KQ. Administrative support: MZ. Provision of study materials or patients: GW and MZ. Collection and assembly of data: YD, CJ, and SY. Data analysis and interpretation: QM, YL, BC, and HW. Manuscript writing: YD. All authors contributed to the article and approved the submitted version.

## Funding

This study was supported in part by the Shantou City Science and Technology Project (CN) (No: 200629235261843) and Young Talents in Higher Education of Guangdong, China (2020KQNCX022).

## Conflict of Interest

The authors declare that the research was conducted in the absence of any commercial or financial relationships that could be construed as a potential conflict of interest.

## Publisher's Note

All claims expressed in this article are solely those of the authors and do not necessarily represent those of their affiliated organizations, or those of the publisher, the editors and the reviewers. Any product that may be evaluated in this article, or claim that may be made by its manufacturer, is not guaranteed or endorsed by the publisher.
